# GRANDCHILDREN WITH DISABILITIES: THE ROLE OF GRANDPARENTS’ RELATIONSHIPS

**DOI:** 10.1093/geroni/igae098.3131

**Published:** 2024-12-31

**Authors:** Kingsley Mbam, Janelle Fassi, Caitlin Coyle, Gary Siperstein, Jeff Ramdass, Jason Rodriquez

**Affiliations:** University of Massachusetts Boston, Boston, Massachusetts, United States; University of Massachusetts Boston, Goffstown, New Hampshire, United States; University of Massachusetts Boston, Boston, Massachusetts, United States; University of Massachusetts Boston, Boston, Massachusetts, United States; University of Massachusetts Boston, Boston, Massachusetts, United States; University of Massachusetts Boston, Boston, Massachusetts, United States

## Abstract

A body of research, largely in the disability and rehabilitation fields, has shown that grandparents support their grandchildren with disabilities (e.g., autism, down syndrome, and physical disability). However, how grandparents’ experiences of helping raise their grandchildren with disabilities influence their identity development in later life is largely unaddressed. This study examined grandparents’ relationships with their grandchildren under 21 with disabilities to learn how their experiences contribute to their identity development and perceptions of aging. We recruited 8 grandparents through senior centers across Massachusetts and conducted in-depth qualitative interviews with them by Zoom or phone. Interview questions asked about grandparents’ aging experiences as they relate to work, family and social relationships and self-perception, detailed questions about the nature of their relationships with grandchildren, identity development, and demographics. After transcribing the interviews, we used the NVivo 14 software program for qualitative data analysis to analyze our data, identify themes, and draw conclusions. We approached analysis using a constant comparative method that sorts data into various groups, according to specified attributes, and then organizes them in a way to formulate new ideas or theory. Themes that emerged were grandparents’ resilience, attitude of gratitude, role conflict of grandparental with parental influence, and the desire to live longer to remain a support for the grandchild. These findings highlight the paradox of grandparents’ emotions in supporting their grandchildren with disabilities for improved quality of life and functioning. Furthermore, these findings have implications for practitioners providing support services to grandparents raising their grandchildren with disabilities.

